# Genomic evidence of pre-invasive clonal expansion, dispersal and progression in bronchial dysplasia

**DOI:** 10.1002/path.2887

**Published:** 2011-06

**Authors:** Frank McCaughan, Christodoulos P Pipinikas, Sam M Janes, P Jeremy George, Pamela H Rabbitts, Paul H Dear

**Affiliations:** 1 MRC Laboratory of Molecular BiologyCambridge, UK; 2 Centre for Respiratory Research, Department of Medicine, Royal Free and University College Medical School, Rayne InstituteLondon, UK; 3 Department of Thoracic Medicine, University College London HospitalsLondon, UK; 4 Leeds Institute of Molecular Medicine, Section of Experimental Therapeutics, St James's University HospitalLeeds, UK

**Keywords:** clonality, bronchial dysplasia, molecular copy-number counting

## Abstract

The term ‘field cancerization’ is used to describe an epithelial surface that has a propensity to develop cancerous lesions, and in the case of the aerodigestive tract this is often as a result of chronic exposure to carcinogens in cigarette smoke 1, 2. The clinical endpoint is the development of multiple tumours, either simultaneously or sequentially in the same epithelial surface. The mechanisms underlying this process remain unclear; one possible explanation is that the epithelium is colonized by a clonal population of cells that are at increased risk of progression to cancer. We now address this possibility in a short case series, using individual genomic events as molecular biomarkers of clonality. In squamous lung cancer the most common genomic aberration is 3q amplification. We use a digital PCR technique to assess the clonal relationships between multiple biopsies in a longitudinal bronchoscopic study, using amplicon boundaries as markers of clonality. We demonstrate that clonality can readily be defined by these analyses and confirm that field cancerization occurs at a pre-invasive stage and that pre-invasive lesions and subsequent cancers are clonally related. We show that while the amplicon boundaries can be shared between different biopsies, the degree of 3q amplification and the internal structure of the 3q amplicon varies from lesion to lesion. Finally, in this small cohort, the degree of 3q amplification corresponds to clinical progression. Copyright © 2011 Pathological Society of Great Britain and Ireland. Published by John Wiley & Sons, Ltd.

## Introduction

The term ‘field cancerization’ was first coined many years ago 1 and the potential underlying biological processes have been studied and discussed at length, particularly in cancers of the aerodigestive tract 2, 3. Three possible mechanisms for field cancerization have been mooted in lung cancer 4. First, an epithelial surface exposed to repeated insult, such as cigarette carcinogens, could develop multiple separate dysplastic foci that do not originate from a single clone but share similar genetic aberrations as a result of the common carcinogenic insult. In time, one or more of these foci may progress to cancer. A second mechanism would be that a mutant clone expands and migrates to colonize the epithelial surface, without breaching the basement membrane, and that molecular divergence results in subclonal populations that may or may not progress. In the third explanation an established cancer spreads to form multifocal clonal tumours. It is also entirely possible that these mechanisms coexist.

In the past shared mutations (particularly of *TP53*) or patterns of loss of heterozygosity (LOH) have been the main biomarkers used to infer the presence or absence of a clonal relationship between separate biopsies from a single individual in lung cancer and pre-invasive bronchial dysplasia 5–9. It could be argued that defining a specific shared mutation in *TP53*, a gene with multiple different mutational hotspots, with or without supportive LOH studies, is more suggestive of clonality than LOH patterns alone 10. In a seminal case report, it was demonstrated that multiple, anatomically distinct biopsies from an individual with widespread mild dysplasia had a common mutation in *TP53*, indicating epithelial colonization at a pre-invasive stage 11. More recently a report on 70 multifocal lung cancers from 30 individuals suggested, on the basis of both LOH and *TP53* mutational analysis, that in 77% of individuals the lesions were clonally related, and concluded that the mechanism underlying their monoclonal origin was local and intrapulmonary metastasis from established cancers 12. An accompanying editorial summarized the current knowledge in this field but noted that it was not possible to exclude pre-invasive clonal migration as an alternative mechanism 4. Therefore, our understanding of the processes underlying bronchial field cancerization remains incomplete.

We have recently demonstrated that a digital PCR technique, microdissection molecular copy-number counting (µMCC), can provide detailed high-resolution information on structural genomic events in archived pre-invasive bronchial biopsies, in which the amount of available tissue for analysis is significantly limited and the DNA is of poor quality 13. We used this approach to show that 3q amplification is consistently observed in high-grade, but not low-grade, bronchial dysplastic lesions, and that the likely focus of this amplification is *SOX2* 14, consistent with recent results from other groups 15, 16.

We now apply the µMCC technique to the question of establishing whether clonal relationships exist between lesions from separate parts of the bronchial tree. We use samples from a longitudinal bronchoscopic study, the Early Lung Cancer Project at UCLH, many of which have already been used in research studies 6, 7, 14, 17. This study was prompted by an observation that at very low resolution (2 Mb) a high-grade lesion and a subsequent cancer appeared to share amplicon boundaries 14. We have now used ultra-high-resolution analysis of shared amplicon boundaries to define clonal relationships between microdissected biopsies taken from anatomically distinct parts of the bronchial tree in the same individuals. This type of approach has been used at lower resolution to infer clonality in cancer 18–22. In this study we apply it to pre-invasive lesions for the first time.

## Materials and methods

### Patients and samples

The patients were enrolled in the University College London Hospital Early Lung Cancer Project (ELCP). This is a longitudinal bronchoscopic surveillance study that has been described previously 17. At the time of enrolment none of the patients had an active diagnosis of lung cancer, although they may have had a prior history of lung cancer. Local Regional Ethical Committee approval was obtained (01/0148). Further details of the methodology and protocol for histological diagnosis have been published 14, 17. Clinical details of the three patients who are the subject of this publication have been published previously 7, 14.

### Microdissection molecular copy-number counting (µMCC)

This protocol has been described recently 13, 23. To summarize, dysplastic epithelium was microdissected from haemotoxylin and eosin (H&E)-stained sections of formalin-fixed paraffin-embedded blocks, using the Arcturus Pixcell II system (Applied Biosystems, USA) in order to minimize any contamination from stroma or normal epithelium. Genomic DNA was extracted from the microdissected cells using the Qiagen DNeasy kit (Qiagen, UK), as previously described 13. Limiting dilution single-molecule PCR was then performed on the template DNA in a two-stage hemi-nested PCR. The first stage is a multiplex reaction, allowing the same template to be simultaneously interrogated for multiple loci; the second a singleplex reaction, assaying each locus individually. For each amplicon boundary, multiple iterative experiments were performed, as previously described 13, to refine boundaries to high resolution. Primers were designed as previously described 13 and all primer sequences are available on request. All genomic loci were from the Ensembl database (http://www.ensembl.org), NCBI Build 36.

### *TP53* sequencing

In the case of one patient, DNA was extracted from biopsies taken at the same bronchoscopy and from the same anatomical location as the lesions used for µMCC. 20 ng DNA was used in a nested PCR protocol for each of exons 5–8 of *TP53*. The primer sequences used are available on request.

## Results

### Case histories

These have been published previously 7, 14 and are therefore summarized briefly here.

#### Case 1 (patient 002)

This patient presented with multifocal left-sided high-grade bronchial dysplasia. She entered the surveillance programme and eventually proceeded to a left lower lobectomy for a radiologically occult stage 1A squamous lung cancer. Twenty-one (HG14) and 13 months (HG1) prior to the left lower lobectomy, bronchoscopies were performed in which biopsies were taken from the left upper and lower lobes.

**Figure 1:**
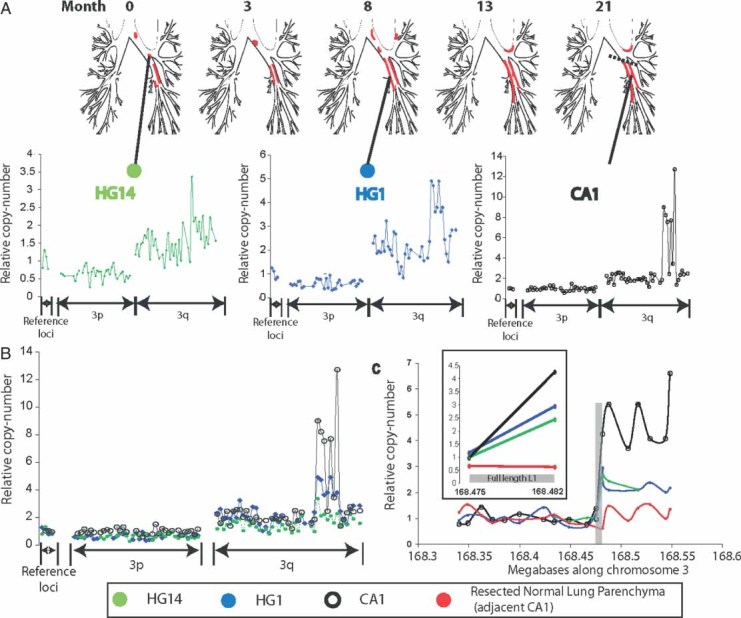
Patient 002—clinical progression is associated with progressive and clonal changes in amplicon structure. (a) Patient 002 had multiple surveillance bronchoscopies over a 21 month period. Comparative µMCC analysis of three lesions was performed, one (HG 14) taken at a bronchoscopy at month 0, another at month 8 (HG1) and the final biopsy (CA1) at month 21. The upper lobe lesion (green) regressed. The lower lobe lesion (blue) progressed to cancer after 13 months (black). Some of the data from HG1 and CA1 have been presented previously 14#. (b) The amplicon differed in structure between the three lesions. µMCC results from the three lesions were compared. There was more pronounced 3q amplification in HG1 that progressed compared to HG14 that did not, and a further increase in 3q amplification in the subsequent cancer CA1. The µMCC profiles suggested that all three lesions shared a centromeric amplicon boundary. (c) Ultra-high resolution µMCC of the transition region was performed. The amplicon boundary was resolved to within a full-length L1 repeat element (locus 168, 475, 750–168, 481, 748) by the use of amplimers that straddled the start and end of the L1 (c, insert). This change in copy number was not present in DNA extracted from lung tissue adjacent to the resected cancer (red) (c). The results were normalized to the mean of the reference loci results for (a) and (b), and to the average of the pre-amplicon markers in (c)

Previously, the proximal 3q amplicon junction was resolved in the stage 1A cancer to an approximately 7 kb interval, spanning a full-length L1 repeat element 13. After repeated rounds of µMCC, it was demonstrated that the boundary lay on either side of a full-length L1 repeat element. We took a similar approach to define the distal boundary of this amplicon and resolved this to an interval of 1.9 kb. The primers used to interrogate the amplicon boundaries in the cancer were then applied to prior biopsies from the same individual (Figure 1). It was readily demonstrated that both the proximal and distal amplicon boundaries were shared between all three biopsies (Figure 1; see also Supporting information, Figure S1). Moreover, there was a marked difference in the amplitude of 3q gain in the separate biopsies. The left lower lobe lesion, which progressed to cancer, had greater 3q amplification than the upper lobe lesion that did not progress. As previously demonstrated for *SOX2* 14, there was an incremental gain in 3q amplification between the high-grade pre-invasive lesion and the cancer, with a preserved amplicon junction. The observation that the cancer (CA1) and the preceding left lower lobe high-grade lesion (HG1) share common amplicon boundaries confirms the clonal evolution of a pre-invasive lesion to an invasive cancer in this patient.

#### Case 2 (patient 017)

This patient underwent a left upper lobectomy in February 2003 for squamous lung cancer (SQC). A subsequent biopsy, taken in 2003 (HG11), and a later resection specimen after completion pneumonectomy showed high-grade pre-invasive disease but no evidence of invasive disease. At the initial post-pneuonectomy bronchoscopy there was no histological evidence of high-grade dysplasia, but at subsequent bronchoscopies HGD was detected in the lower trachea (HG5) and a later biopsy from the pneumonectomy stump showed invasive cancer (CA2).

**Figure 2:**
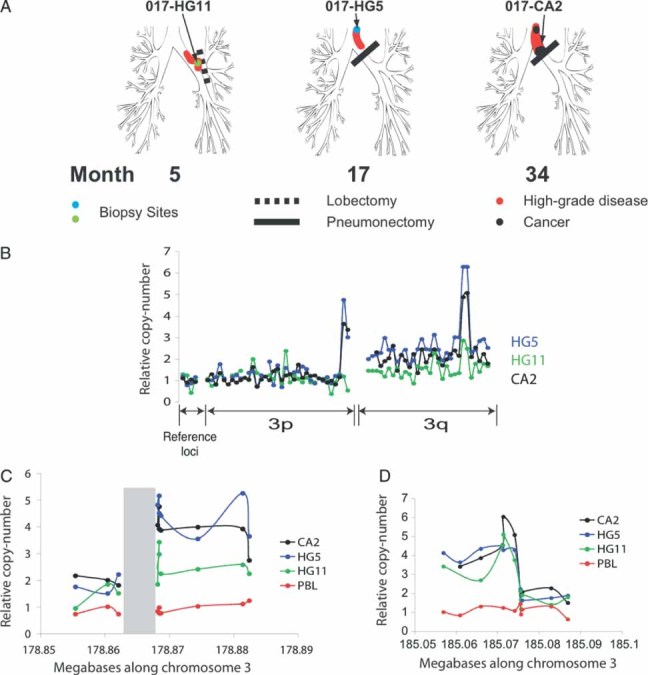
Patient 017—clinical progression is associated with progressive and clonal changes in amplicon structure (a) Comparison of sequential bronchial biopsies from patient 017, who underwent a left upper lobectomy (dashed bar) for squamous cell carcinoma prior to entering the study. He was subsequently found to have high-grade dysplasia in the resection margin and had a number of surveillance bronchoscopies. Although there was no biopsy-proven evidence of invasion, there was a clinical suspicion of cancer, so he proceeded to a pneumonectomy (solid bar) at month 15. The pneumonectomy specimen confirmed high-grade pre-invasive disease with no focal invasion. Subsequent biopsies at 17 and 34 months demonstrated extensive involvement of the trachea and two foci of invasion were diagnosed, including one at the pneumonectomy stump. Local photodynamic therapy was undertaken but the patient died of complications after the procedure. Three biopsies were analysed. (b) µMCC results for chromosome 3 are shown for all three biopsies and demonstrate regional amplification between 178 and 185 Mb. Using the low-resolution markers there is an increase in 3q amplification of the later lesions (HG5 and CA2) compared to HG11, confirmed on higher resolution junction analysis as shown in (c). Data from HG5 has been published previously 14#. (c, d) Iterative µMCC experiments were performed to define the centromeric and telomeric amplicon boundaries for each biopsy, using DNA derived from peripheral blood leukocytes (PBLs) of the same individual as a control. There was a common telomeric boundary for all three biopsies to a resolution of 1416 bps in the interval 185, 077, 260–185, 078, 676 within introns 1–2 of the gene *PARL*. The centromeric boundary again lay within an L1 element for the two later lesions. HG11 did not share the centromeric boundary; with similar copy pre- and post- the L1 repeat element, however, there was evidence of an adjacent amplicon boundary

Low-resolution µMCC of HG5 performed previously revealed a focal amplicon distally on 3q 14. The boundaries of this amplicon were now defined at high resolution and a similar analysis was performed on all three specimens. The three epithelial biopsies had a common telomeric amplicon boundary, indicating a clonal origin (Figure 2). The later biopsies had both developed a focal precentromeric amplicon on 3p. Interestingly, although the 3q amplicon from both of the later biopsies shared a centromeric boundary, again within a full-length L1 element, the earlier lesion had an adjacent but not identical boundary. In this patient there was sufficient DNA available from diagnostic biopsies, taken at the same time and from the same anatomical sites as the research biopsies HG11 and CA2, to perform *TP53* mutational analysis. In both biopsies there was a common mutation in exon 5 of *TP53* (see Supporting information, Figure S2), again consistent with these lesions having a clonal origin.

**Figure 3:**
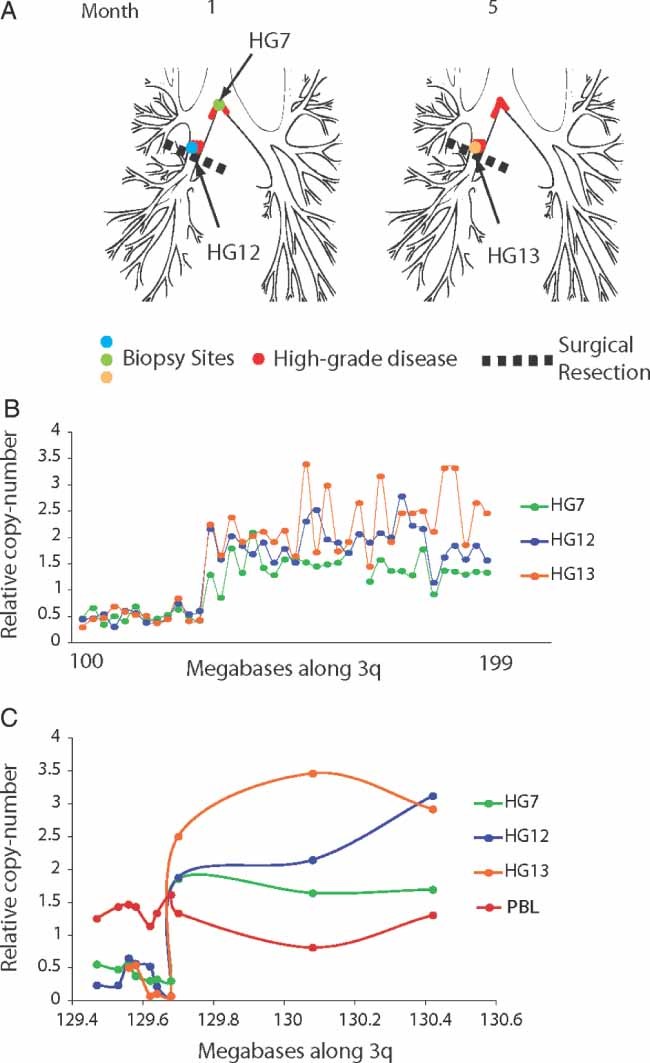
Patient 026—clinical progression is associated with progressive and clonal changes in amplicon structure. (a,# b) Comparison of three biopsies from patient 026, who entered the surveillance programme after surgical resection of the right middle and lower lobes for SQC; two biopsies (HG7 and 12) taken at the first surveillance bronchoscopy, and another (HG13) at a subsequent bronchoscopy at month 5. Photodynamic therapy was performed with good effect after a diagnosis of microinvasive cancer at the same anatomical site as HG12 and HG13 in month 13. The µMCC data from chromosome 3 suggest an incremental increase in 3q amplification between HG12 and HG13, and they also suggest that all three biopsies share a common centromeric amplicon boundary. (c) On high-resolution analysis, this was confirmed to lie in the interval 129, 680, 327–129, 700, 853 bps, with the results suggesting deletion of a genomic segment immediately proximal to the amplicon

#### Case 3 (patient 026)

Patient 026 had a successful right lower lobe lobectomy for SQC. At the post-operative surveillance bronchoscopy there was high-grade dysplasia near the lobectomy stump in the bronchus intermedius. In addition, a ‘control’ biopsy from the main carina also showed high-grade dysplasia. HGD of the bronchus intermedius (HG12,13) progressed to microinvasive cancer, whereas at the main carina (HG7) it did not. The structure of the 3q amplicon in this patient was different, with a clear centromeric boundary, but in this case the amplicon extended to the telomere. µMCC analysis of the centromeric amplicon boundaries of the three biopsies was consistent with their sharing a monoclonal origin. We have previously demonstrated progressive *SOX2* amplification in the two bronchus intermedius samples from this patient (HG12 and 13) 14. The current data confirm progressive amplification of the whole amplified 3q segment in the course of clinical progression. The common centromeric amplicon boundary is retained but the amplitude and internal structure of the 3q amplicon changes. It is again noteworthy that the main carina dysplastic lesion, which did not have the more marked 3q amplification seen in the lesions from the bronchus intermedius, did not progress.

## Discussion

We have used µMCC to closely interrogate regional genomic events in archived microdissected bronchial biopsies. Field cancerization in the bronchial tree is often described, both in clinical practice and in terms of molecular biomarkers, but the mechanism and natural history of this process remains unclear. One question is whether dysplastic bronchial cells can migrate and disperse intra-epithelially to colonize the epithelial layer prior to breaching the basement membrane. The three patients discussed had no evidence of invasive lung cancer (radiological or otherwise) at the point of entry into the longitudinal study. Patient 002 did not have invasive disease prior to enrolment and, although the others did, all three are instructive.

The first finding is that in patients without overt lung cancer, a precisely resolved amplicon boundary can be used to define a monoclonal origin of pre-invasive bronchial biopsies that are both anatomically separate and molecularly heterogeneous. It has been suggested before that such boundaries, resolved at lower resolution, can be viewed as markers of clonality 18. Although many cancers are characterized by regional amplification encompassing particular genes, the specific structure of these events varies from individual cancer to individual cancer. With respect to 3q amplification, the scale of the region involved is often many megabases in size. There are no data to suggest a specific ‘hot spot’ for 3q amplification in squamous lung cancer. The evidence from our previous studies and those of others suggest that the structure and boundaries of the 3q amplicon varies tremendously between lesions from different individual patients 14–16, 24. Therefore, amplicon boundaries common to separate biopsies from a single individual could be regarded as personalized markers of clonality. Further, for patient 002, both centromeric and telomeric boundaries are shared. These shared biomarkers in the three patients presented indicate a clonal origin for the separate biopsies in each individual and are consistent with a previous case report of widespread clonal dissemination prior to invasion 11. The use of amplicon boundaries to infer clonality is also supported by the data from Patient 017, that both lesions tested shared a common somatic mutation in *TP53*. The involvement of repeat elements in amplicon boundaries is consistent with what others have shown in genome-wide studies of structural rearrangements in invasive cancer 25.

These data, using precisely delineated amplicon boundaries as genomic biomarkers, confirm that clonal expansion and dispersal of cells occurs and that subclonal populations emerge with varying molecular characteristics and clinical outcomes. The lesions studied, with the exception of HG11 and CA2 in case 2, were distinct in terms of their chromosome 3 profile, consistent with the existence of intra-epithelial subclonal diversity. This is interesting in the context of recent work on Barrett's oesophagus, in which measures of clonal diversity were robust predictors of progression to cancer 26. Although this is a small cohort, the other consistent observation in the current cases is that the amplitude of 3q amplification predicts which lesion is likely to progress; and further, that there is incremental 3q amplification in those lesions that do progress. The progression of dysplasia to invasion is generally held to reflect ongoing selection of clonally advantageous genetic events. However, the contribution of regional genomic events to neoplastic progression is a focus of some debate—whether it is of pathogenic consequence or an epiphenomenon reflecting genome instability. 3q amplification may be selectively neutral or it may be a critical stage in the development of squamous lung cancer. In support of the latter, the almost uniform finding of 3q amplification in invasive squamous lung cancer 27 and its selection in the progression to invasion points to a significant and perhaps obligate role in the pathogenesis of SQC. Whether *SOX2* is the main target of this amplification or one of a number of target oncogenes that are co-amplified remains unclear.

This is a short case series, reflecting the difficulty in recruiting to this type of longitudinal bronchoscopic study. However, as with prior individual case reports 5, 11, but this time using an individualized genomic biomarker, this provides convincing evidence that widespread pre-invasive clonal dispersal is one mechanism underpinning field cancerization in the lung. This work demonstrates the potential value of longitudinal studies; there is a pressing need for similar larger-scale studies, particularly in high-risk individuals who do not have a history of lung cancer.

## Footnotes

# Please note that versions of Figures 1a, 2a and 3a that illustrate the patients' clinical history and some of the associated µMCC data have been published previously 7, 14. Reprinted with permission of the American Thoracic Society. Copyright © American Thoracic Society.

## Supporting Information on the Internet

The following supporting information may be found in the online version of this article

**Figure S1.** Distal 3q amplicon boundary is shared by three lesions. Using an iterative approach, the distal amplicon boundary was resolved to a 1.9 kb interval and was the same for the three lesions, HG1, HG14 and CA1.

**Figure S2.** Sequencing of exon 5 of *TP53* was performed on DNA extracted from three bronchial biopsies from patient 017.
