# Supplementary figures and images for: Genomic evidence of pre-invasive clonal expansion, dispersal and progression in bronchial dysplasia
Source: J Pathol. 2011 Jun;224(2):153–9. doi: 10.1002/path.2887 (PMC3378694; doi:10.1002/path.2887)

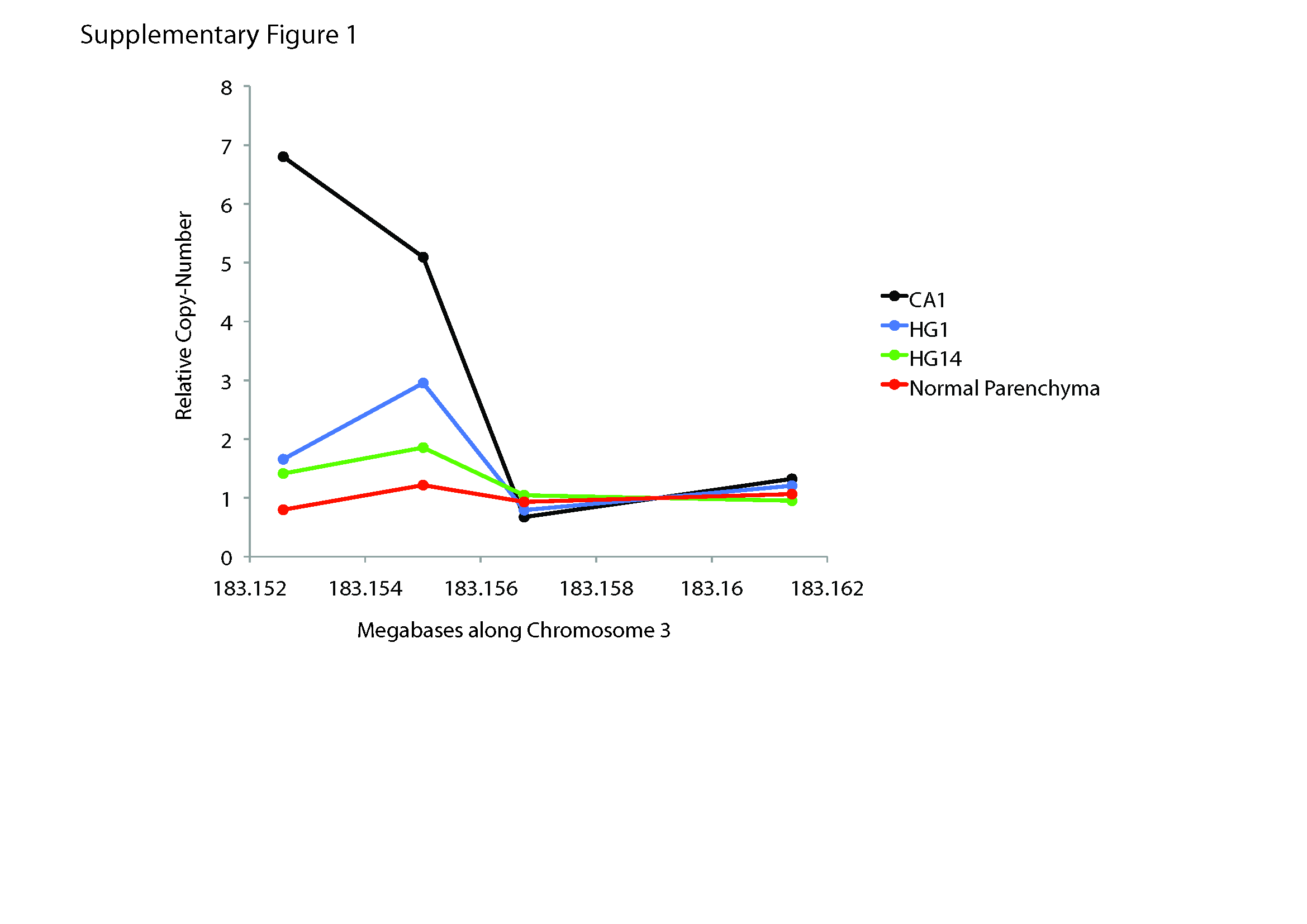

Supplement: Supplementary file 1 [file path0224-0153-SD1.tiff]

Supplementary Figure 2

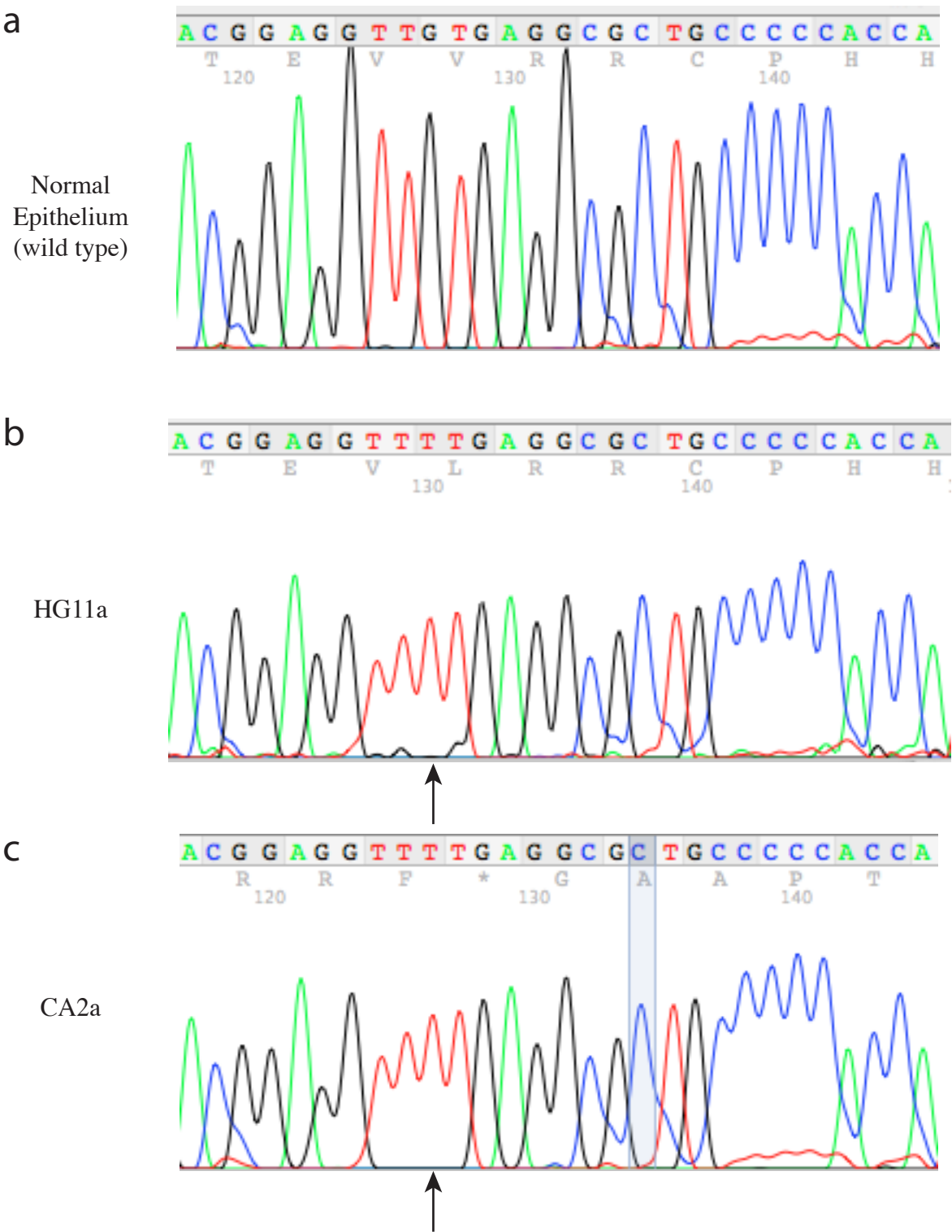

Supplement: Supplementary file 2 [file path0224-0153-SD2.pdf]
